# Supplementary material for: Iterative improvement in the automatic modular design of robot swarms
Source: PeerJ Comput Sci. 2020 Dec 7;6:e322. doi: 10.7717/peerj-cs.322 (PMC7924708; doi:10.7717/peerj-cs.322)
Supplement: Supplemental Information 3 [file peerj-cs-06-322-s003.zip › argos3/doc/api/standalone/a00476_source.html]

ARGoS: plugins/robots/foot-bot/simulator/footbot\_turret\_encoder\_default\_sensor.cpp Source File


- Main Page
- Related Pages
- Namespaces
- Classes
- Files

- File List
- File Members

# plugins/robots/foot-bot/simulator/footbot\_turret\_encoder\_default\_sensor.cpp

Go to the documentation of this file.

```
00001 
00007 #include "footbot_turret_encoder_default_sensor.h"
00008 #include <argos3/core/simulator/entity/composable_entity.h>
00009 
00010 namespace argos {
00011 
00012    /****************************************/
00013    /****************************************/
00014 
00015    CFootBotTurretEncoderDefaultSensor::CFootBotTurretEncoderDefaultSensor() :
00016       m_pcTurretEntity(NULL) {}
00017 
00018    /****************************************/
00019    /****************************************/
00020 
00021    void CFootBotTurretEncoderDefaultSensor::SetRobot(CComposableEntity& c_entity) {
00022       m_pcTurretEntity = &(c_entity.GetComponent<CFootBotTurretEntity>("turret"));
00023       m_pcTurretEntity->Enable();
00024    }
00025 
00026    /****************************************/
00027    /****************************************/
00028 
00029    void CFootBotTurretEncoderDefaultSensor::Update() {
00030       m_cRotation = m_pcTurretEntity->GetRotation();
00031    }
00032 
00033    /****************************************/
00034    /****************************************/
00035 
00036    void CFootBotTurretEncoderDefaultSensor::Reset() {
00037       m_cRotation = CRadians::ZERO;
00038    }
00039 
00040    /****************************************/
00041    /****************************************/
00042 
00043    REGISTER_SENSOR(CFootBotTurretEncoderDefaultSensor,
00044                    "footbot_turret_encoder", "default",
00045                    "Carlo Pinciroli [ilpincy@gmail.com]",
00046                    "1.0",
00047                    "The foot-bot turret encoder sensor.",
00048                    "This sensor accesses the foot-bot turret encoder. For a complete\n"
00049                    "description of its usage, refer to the ci_footbot_turret_encoder_sensor\n"
00050                    "file.\n\n"
00051                    "REQUIRED XML CONFIGURATION\n\n"
00052                    "  <controllers>\n"
00053                    "    ...\n"
00054                    "    <my_controller ...>\n"
00055                    "      ...\n"
00056                    "      <sensors>\n"
00057                    "        ...\n"
00058                    "        <footbot_turret implementation=\"default\" />\n"
00059                    "        ...\n"
00060                    "      </sensors>\n"
00061                    "      ...\n"
00062                    "    </my_controller>\n"
00063                    "    ...\n"
00064                    "  </controllers>\n\n"
00065                    "OPTIONAL XML CONFIGURATION\n\n"
00066                    "None for the time being.\n",
00067                    "Usable"
00068       );
00069 
00070 }
```

---

Generated on 10 Jul 2018 for ARGoS by 
 1.6.1
